# Job Strain, Job Insecurity, and Incident Cardiovascular Disease in the Women’s Health Study: Results from a 10-Year Prospective Study

**DOI:** 10.1371/journal.pone.0040512

**Published:** 2012-07-18

**Authors:** Natalie Slopen, Robert J. Glynn, Julie E. Buring, Tené T. Lewis, David R. Williams, Michelle A. Albert

**Affiliations:** 1 Center on the Developing Child, Harvard University, Cambridge, Massachusetts, United States of America; 2 Department of Society, Human Development, and Health, Harvard School of Public Health, Boston, Massachusetts, United States of America; 3 Department of Preventive Medicine, Brigham and Women’s Hospital, Harvard Medical School, Boston, Massachusetts, United States of America; 4 Department of Epidemiology and Public Health, Yale University School of Medicine, New Haven, Connecticut, United States of America; 5 Department of African and African American Studies, Harvard University, Cambridge, Massachusetts, United States of America; 6 Division of Cardiovascular Medicine, Brigham and Women’s Hospital, Harvard Medical School, Boston, Massachusetts, United States of America; University of Bochum, Germany

## Abstract

**Objectives:**

Research about work-related stressors and cardiovascular disease (CVD) has produced mixed findings. Moreover, a paucity of data exists regarding the long-term associations between job strain and job insecurity and CVD among women.

**Methods:**

We used Cox proportional hazard models to examine the relationship between job strain, job insecurity, and incident CVD over 10 years of follow-up among 22,086 participants in the Women’s Health Study (mean age 57±5 years).

**Results:**

During 10 years of follow-up there were 170 myocardial infarctions (MI), 163 ischemic strokes, 440 coronary revascularizations, and 52 CVD deaths. In models adjusted for age, race, education, and income, women with high job strain (high demand, low control) were 38% more likely to experience a CVD event than their counterparts who reported low job strain (low demand, high control; Rate Ratio (RR) = 1.38, 95% Confidence Interval (CI) = 1.08–1.77), and women with active jobs (high demand, high control) were 38% more likely to experience a CVD event relative to women who reported low job strain (95% CI = 1.07–1.77). Outcome-specific analyses revealed that high job strain predicted non-fatal myocardial infarction (RR = 1.67, CI = 1.04–2.70), and coronary revascularization (RR = 1.41, CI = 1.05–1.90). No evidence of an association between job insecurity and long-term CVD risk was observed.

**Conclusion:**

High strain and active jobs, but not job insecurity, were related to increased CVD risk among women. Both job strain and job insecurity were significantly related to CVD risk factors. With the increase of women in the workforce, these data emphasize the importance of addressing job strain in CVD prevention efforts among working women.

## Introduction

Cardiovascular disease (CVD) is the leading cause of death for both women and men in developed [1] and developing [2] nations. Extensive research provides evidence that psychosocial stressors increase the risk for CVD, including hypertension, myocardial infarction (MI), atherosclerotic disease, and stroke [3], [4]. The INTERHEART case-control study of 24,767 individuals from 52 countries demonstrated that psychosocial stress related to work, finances, home, and life events was associated with a 2-fold increased risk of acute MI, accounting for a population risk attributable to chronic stress of 33% [5]. Among studied psychosocial stressors, job-related stressors have received considerable research attention [6]–[8], however prospective data in women are sparse [9]. Job strain – a two-dimensional model for psychosocial work conditions that considers demands and control [10] – and job insecurity, which reflects concern about future job loss, are two work-related psychosocial stressors that have been examined as risk factors for a variety of health outcomes. Although inconsistencies exist [11], [12], the majority of evidence from case-control and cohort studies of men supports associations between job-related stressors and incident CVD [6], [7], [13]. A recent systematic review of prospective studies concluded that there is moderate evidence that psychosocial factors at work are related to CVD, but that findings among women are not clear [14].Further knowledge about the effects of job-related stressors on cardiovascular health among women is important, given the dramatic increase in female participation in the labor force over the past several decades, and that psychosocial stressors may affect women and men in distinct ways.

Among existing studies of job-related stressors and CVD that have included women, there is limited and inconsistent evidence regarding the relationship between high job strain [12], [15]–[17] and job insecurity [18], [19] and CVD. In the Nurses Health Study, a large prospective cohort of 35,000 female nurses, job strain was not associated with incident coronary heart disease (CHD) over 4 years of follow-up [16], although job insecurity was related to risk of non-fatal MI over 2-years, but not over a 4-year follow-up period [18]. An examination of 48,361 women in Finland (ages 18 to 65) revealed that active jobs (i.e., characterized by high demands and high control) were associated with over 2 times the risk of incident cerebrovascular disease over 3-years of follow-up [20].

We examined the relationship between job strain and job insecurity and incident CVD among participants in the Women’s Health Study (WHS), a prospective cohort of female health professionals recruited from across the entire United States. We hypothesized that high job strain and job insecurity would predict incident CVD over 10 years of follow-up.

## Methods

### Participants

Study subjects were participants in the WHS, a randomized, placebo-controlled trial of aspirin and vitamin E for the primary prevention of CVD and cancer among apparently healthy female health professionals (75% registered nurses, 15% licensed practical/visiting nurses, 2.5% medical doctors, 8% other health professionals; ClinicalTrials.gov Identifier: NCT00000479) [21], [22]. Details of WHS study design have been previously described [21], [22]. In brief, invitation letters to participate in the baseline questionnaire and trial were mailed to over 1.7 million female health care professionals across 50 US states and the District of Columbia. Of the 453,787 women who completed the questionnaire, 65,169 women were eligible to participate (based on age, disease history, medication history; see other publications for details [21], [22]). Following a 3-month run-in period to identify participants likely to comply with the study protocol, 39,876 women were randomized (February 1993) and participants were followed until March 2007 (see **Figure S1**). Socio-demographic baseline variables were collected using mailed questionnaires. At the conclusion of the trial, follow-up questionnaires to assess a variety of health outcomes were sent every 6 months during the first year and yearly thereafter. Job-related stressors were assessed via mail questionnaire in Year 5 (baseline for this analysis). Demographic and clinical data were also collected in Year 5, including: age, race/ethnicity, education, income, employment and marital status; parental history of MI before 60 years, menopausal status, smoking status, alcohol consumption, physical activity, body mass index (BMI), history of hypertension, type II diabetes (diabetes),and hypercholesterolemia; and, symptoms of depression and anxiety in the past 4 weeks, measured using the RAND Medical Outcomes Survey 4-item scale [23]. Women were followed for 10 years.

Women were eligible for the present study if they participated in the Year 5 (1998) questionnaire and were without known cardiovascular disease at this time (N = 34,717). Job stress questions were only requested to be completed by women who had been employed within 2 years prior to the questionnaire; a total of 10,883 women did not have responses to the job stress items and therefore were excluded (N = 23,834). Some participants were retired, full-time homemakers, or disabled at the time of the work stress questions, yet were eligible to report on their work stress if they had been employed in the 2-year period prior to questionnaire administration. In order to qualify for the main analyses, we required that respondents had complete information on job stressors, CVD outcomes, age, race, education and income (N = 22,086). See **Table S1** for comparison of baseline characteristics for participants in main analysis (N = 22,086) and participants excluded from main analysis due to missing work stress questions (N = 10,833) or covariates of age, race, education, income (N = 1748). Women who did not have completed work stress items were on average 4.5 years older than women with complete data on the work stress items, and differed on baseline characteristics in a manner consistent with older age. There were not systemic differences between women included in the main analysis and those excluded due to missing data on age, race, education, or income.

We carried out a sub-analysis to examine potential mediators and additional confounders on women in the main analyses who had complete data on a broad set of demographic and health variables (N = 17,415). See **Table S2** for comparison of women included in this sub-analysis (i.e., complete data on additional covariates; N = 17,415) and participants excluded from sub-analysis due to missing data. The Institutional Review Board of Brigham and Women’s Hospital approved the trial and ongoing observational follow-up, and women provided written informed consent.

### Psychosocial Job Characteristics

A measure of job strain was derived from Karasek’s Job Content Questionnaire [24], which assessed job demand and job control using 14 Likert-style items. Each item had 4 response options: strongly disagree, disagree, agree, and strongly agree. The job demand scale was comprised of 5 items which ask about the pace, challenge, and amount of work, time to complete work, and conflicting demands. The job control scale was a composite of two sub-scales: decision authority (3 items) and skill discretion (6 items). The decision authority subscale measures autonomy in decision making, freedom, and authority. The skill discretion subscale measures whether the job requires creativity, learning, repetition, high skill, variation, and opportunities to develop abilities (see **Table S3** for list of items). The demand and control scales were dichotomized based on their median scores, and women were assigned to 1 of 4 categories according to scores on each dimension: passive (low demand and low control), active (high demand and high control), low strain (low demand and high control), or high strain (high demand and low control) [24].

A measure of job insecurity was derived from responses to the question: “My job security is good”. Response options ranged from (1) strongly disagree to (4) strongly agree. Following previous research using this item [18], we categorized responses of strongly disagree and disagree as job insecure, and responses of agree or strongly agree as job secure.

### Cardiovascular Disease End Points

Cardiovascular events included non-fatal myocardial infarction (MI), non-fatal ischemic stroke, revascularization procedure (coronary artery bypass grafting and/or percutaneous transluminal coronary angioplasty), and CVD death. Outcomes were reported via mail questionnaires, letters, and telephone calls. Information about deaths was acquired from the National Death Index, or reports from family members or the postal service. Medical records were ascertained from physicians and hospitals after women or next of kin provided consent, and blinded physicians in the WHS Independent Endpoints Committee reviewed records to confirm symptoms met criteria for each outcome. MIs were confirmed according to criteria specified by World Health Organization, as well as diagnostic ECG criteria or abnormal levels of cardiac enzymes. Ischemic strokes were confirmed if symptoms were consistent with a new neurological deficit that lasted more than 24 hours; computed tomographic scans and magnetic resonance images were used to differentiate ischemic and hemorrhagic strokes. Coronary revascularization procedures were confirmed based on review of hospital records, while CVD deaths were confirmed based on reviews of death certificates, autopsy reports, family reports, and medical records [25]. We included only events confirmed by medical record review. Consent for confirmation was received from 96% of reported events (based on combined information from MI (97%), Stroke (96%), CABG (98%), PTCA (95%), CVD Death (99%)).

**Figure 1:**
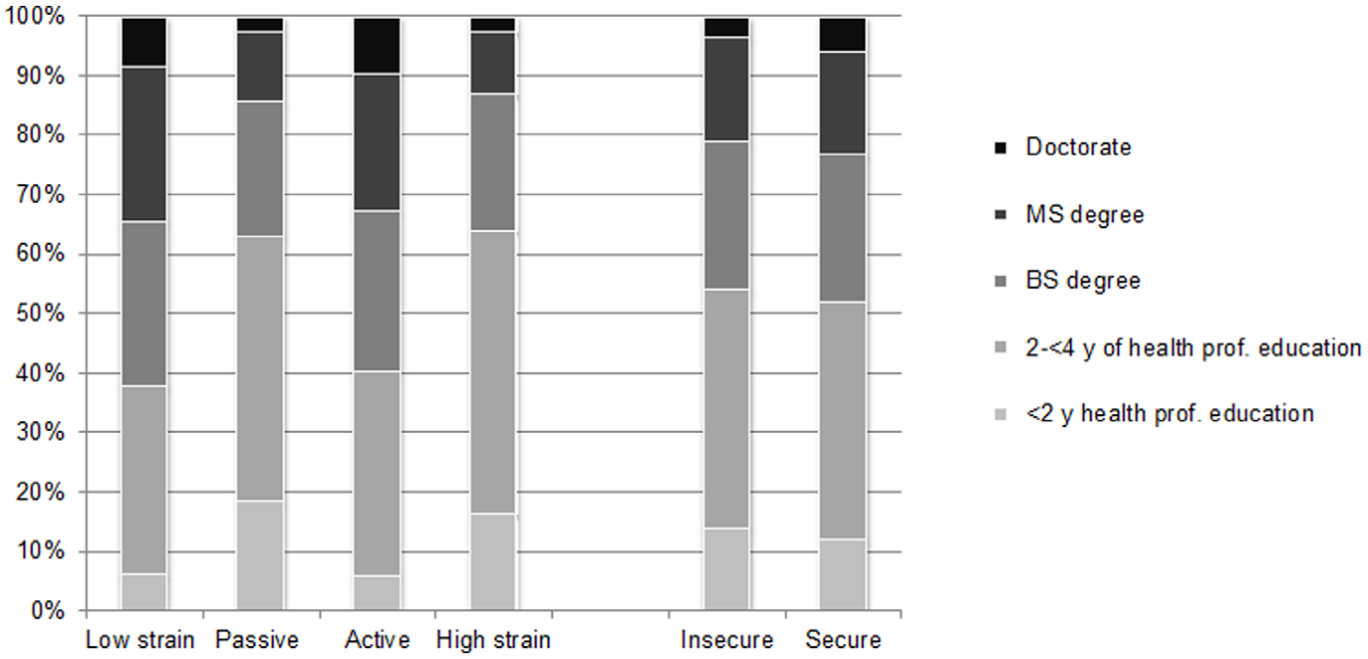
Education Level according to Job Strain and Job Insecurity Categories among Women Health Study Participants (N = 22,086).

**Table 1:** Cardiovascular risk factors and other characteristics according to job strain and job insecurity*.

Job Characteristics	Full Sample	Job Strain Categories					Job Insecurity		
		Low strain	Passive	Active	High strain	*p*-value	Insecure	Secure	*p*-value
		(low demand, high control)	(low demand, low control)	(high demand, high control)	(high demand, low control)				
**Main Sample (N = 22,086)**									
Full sample, N (%)	–	5261 (23.8)	7604 (34.4)	4716 (21.4)	4505 (20.4)		4287 (19.4)	17799 (80.6)	
Job insecure (%)	–	11.5	18.4	17.7	32.2	<.0001	–	–	–
Age, years (Mean, SD)	57.2 (5.2)	57.3 (5.1)	58.3 (5.8)	56.1 (4.2)	56.7 (4.6)	<.0001	56.9 (4.9)	57.3 (5.2)	<.0001
Education (%)									
<2 y health prof. education	12.5	6.2	18.5	6.1	16.4	<.0001	14.1	12.1	<.0001
2-<4 y of health prof. education	40.0	31.7	44.6	34.3	47.7		40.1	39.9	
BS degree	24.8	27.7	22.6	26.9	22.8		24.9	24.8	
MS degree	17.3	26.1	11.8	23.0	10.5		17.4	17.3	
Doctorate	5.4	8.3	2.5	9.7	2.6		3.6	5.9	
Household Income (%)									
<$19,000	2.9	1.7	4.8	1.0	2.9	<.0001	3.9	2.6	<.0001
$20-29,999	7.5	4.9	10.9	3.8	8.8		9.1	7.1	
$30-39,999	12.9	10.8	15.0	9.7	15.4		15.7	12.3	
$40-49,999	17.0	15.2	18.0	15.5	18.8		18.5	16.6	
$50-99,999	46.2	49.5	41.5	52.5	43.7		42.4	47.1	
≥$100,000	13.5	17.8	9.8	17.5	10.4		10.5	14.2	
**Sub-analysis Sample (N = 17,415)**									
Hypertension (%)	37.8	35.9	40.4	34.5	39.1	<0.001	41.2	37.0	<0.001
Diabetes mellitus (%)	4.0	3.5	4.4	3.6	4.3	0.07	5.2	3.7	<0.001
Hypercholesterolemia (%)	42.0	39.2	44.7	39.7	43.0	<0.001	43.7	41.6	0.02
Depressive/anxious symptoms (mean, SD)	10.5 (3.6)	9.5 (3.1)	10.3 (3.5)	10.6 (3.5)	11.8 (4.1)	<0.001	11.7 (4.2)	10.2 (3.4)	<0.001
BMI(kg/m^2^) (mean, SD)	27.0 (5.4)	27.0 (5.3)	27.1 (5.4)	27.0 (5.5)	27.2 (5.6)	0.33	27.6 (5.8)	26.9 (5.3)	<0.001
Obese (BMI>30)	24.5	23.4	24.9	24.5	25.1	0.25	27.9	23.7	<0.001
Physical activity (%)									
Rare/never	36.4	33.3	39.8	32.2	38.6	<0.001	38.4	35.9	0.02
<1× a week	20.7	20.8	20.2	21.4	20.9		20.8	20.7	
1−3× a week	32.5	33.3	30.7	35.0	32.2		30.8	32.9	
4× a week	10.4	12.6	9.4	11.4	8.4		10.0	10.5	
1+ Alcoholic drink/day (%)	10.4	12.6	8.9	11.3	9.2	<0.001	9.7	10.5	0.18
Current smoker (%)	10.8	9.5	10.8	9.9	13.3	<0.001	12.8	10.3	<0.001
Employment (%)									
Full time or part time	89.5	91.2	88.0	91.3	88.0	<0.001	85.9	90.3	<0.001
Full time home maker/ Retired/ Not employed	10.2	8.8	11.7	8.4	11.2		13.2	9.5	
Disabled	0.3	0.1	0.3	0.3	0.7		0.9	0.2	
Marital status (%)									
Single	5.9	6.0	5.5	6.4	5.9	<0.001	6.8	5.7	<0.001
Currently married	74.2	75.5	74.1	74.1	72.7		68.1	75.6	
Divorced or separated	15.5	14.6	14.9	16.1	16.6		20.1	14.4	
Widowed	4.5	3.9	5.5	3.3	4.8		5.0	4.4	
Parental history of MI before 60 yrs (%)	13.5	13.6	12.6	13.7	14.6	0.05	14.5	13.3	0.07

* Values are mean (SD), or percent where indicated.

BMI=body mass index MI = myocardial infarction.

This sample is reduced to women with complete data on an extensive set of health and demographic covariates (used in Models 3 and 4 N=17,415). For this subset of women, the mean levels and frequencies of age, education, and income according to job strain and insecurity category are presented in Table S5.

### Statistical Analysis

Baseline characteristics of the study population are reported as means and frequencies, stratified by category of job strain and job insecurity; significant differences were tested using ANOVA or chi-square statistic. Relative rates of CVD were estimated using Cox proportional-hazards models. Separate models were used to evaluate job strain and insecurity; low job strain and high job security were the reference categories, respectively. For each of the 4 CVD endpoints, as well as a composite outcome comprising the first occurrence of any endpoint, a series of 4 models were calculated. Individuals were censored at time of CVD event or at the end of follow-up period. Model 1 adjusted for age, race (non-Hispanic White, Hispanic, African American/Black, Asian/Pacific Islander, American Indian/Alaskan Native, Other), and randomized study drug assignment (standard practice for studies of the WHS observational follow-up). Model 2 adjusted for variables in Model 1, plus variables to control for socioeconomic status (education and median annual household income).

**Table 2:** Hazard rate (HR) and 95% confidence interval of cardiovascular disease by job strain (N = 22086).

	Low strain	Passive	Active	High strain
	(low demand, high control)	(low demand, lowcontrol)	(high demand,high control)	(high demand, lowcontrol)
	N = 5261	N = 7604	N = 4716	N = 4505
**Total CVD**				
Events (Total N = 661)	118	266	126	151
Model 1	1.00	**1.37 (1.10, 1.70)**	**1.39 (1.08, 1.79)**	**1.63 (1.28, 2.08)**
Model 2	1.00	1.16 (0.93, 1.45)	**1.38 (1.07, 1.77)**	**1.38 (1.08, 1.77)**
**Myocardial infarction**				
Events (Total N = 170)	29	69	28	44
Model 1	1.00	1.47 (0.95, 2.28)	1.20 (0.71, 2.02)	**1.88 (1.18, 3.01)**
Model 2	1.00	1.31 (0.84, 2.05)	1.21 (0.72, 2.03)	**1.67 (1.04, 2.70)**
**Ischemic stroke**				
Events (Total N = 163)	28	68	28	39
Model 1	1.00	1.39 (0.89, 2.16)	1.39 (0.82, 2.35)	**1.83 (1.12, 2.97)**
Model 2	1.00	1.12 (0.71, 1.76)	1.35 (0.80, 2.29)	1.43 (0.87, 2.34)
**Coronary revascularization**				
Events (Total N = 440)	82	173	81	104
Model 1	1.00	**1.30 (1.00, 1.69)**	1.25 (0.92, 1.71)	**1.59 (1.19, 2.13)**
Model 2	1.00	1.14 (0.87, 1.50)	1.25 (0.92, 1.70)	**1.41 (1.05, 1.90)**
**Cardiovascular death**				
Events (Total N = 52)	12	19	12	9
Model 1	1.00	0.89 (0.43, 1.85)	1.55 (0.68, 3.49)	1.07 (0.45, 2.55)
Model 2	1.00	0.68 (0.32, 1.44)	1.59 (0.70, 3.61)	0.84 (0.35, 2.06)

95% confidence intervals shown in parentheses;

Model 1 is adjusted for age; race; and study drug randomization.

Model 2 is adjusted for covariates in Model 1, in addition to education (<2 y health profession education; 2-<4 y health professional education; bachelor’s degree; master’s degree; doctorate); income (<$19,000; $20,000–29,999; $30,000–39,999; $40,000–49,999; $50,000–99,999; ≥$100,000).

Coronary revascularization includes coronary artery bypass grafting and percutaneous transluminal coronary angioplasty.

In a sub-analysis among women with complete data on a larger set of health and demographic variables (N = 17,415), we ran two additional models. Model 3 included the covariates in Model 2, plus depression and anxiety symptoms. Model 4 adjusted for variables in Model 3, plus traditional coronary risk factors, including smoking status, alcohol intake, BMI, history of hypertension, diabetes, hypercholesterolemia, frequency of physical activity (4 categories), parental history of MI before age of 60 years, marital status, current work status (full or part time; retired or full time home maker; disabled), and menopausal status. Since both psychological and traditional risk factors may be part of the causal pathway between work-related stressors and CVD [11], we considered Model 2 as the most parsimonious model. We examined the proportional hazards assumption for Model 2 by including covariates for the interaction between job strain/job insecurity and the logarithm of time in the models.

**Figure 2:**
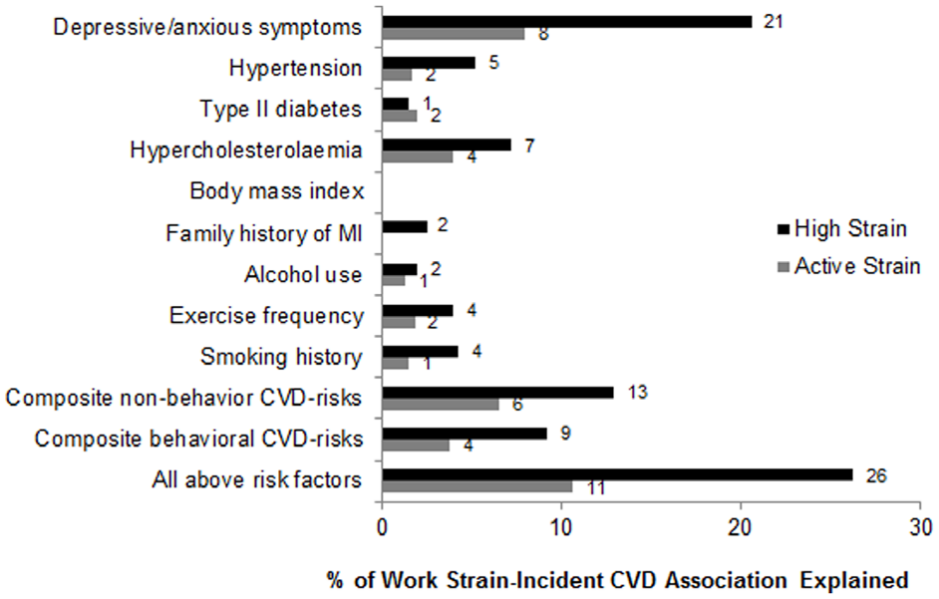
Percentage of work strain-incident CVD associations that are explained by potential mediators (N = 17,415). The proportion of the risk attributable to high strain and active jobs (relative to low strain) that is explained by each mediator (or set of mediators) is calculated as follows: (HR_base model_ – HR_adjusted model_)/ (HR_base model_ –1)×100%. ^1^Composite of traditional non-behavioral risk factors includes hypertension, diabetes, hypercholesterolaemia, body mass index, and parental history of MI before age of 60 years. ^2^Composite of behavioral risk factors includes alcohol use, exercise frequency, and smoking history.

We performed sensitivity analyses where we replicated Models 1 to 4 to evaluate job demand and control separately (using median splits, tertiles, and continuous scores). We also tested for any potential interaction between job strain and job insecurity with total CVD risk. Finally, in the subset of women with complete data on the health and demographic factors, we evaluated the contribution of each individual risk factor (as well as groupings of measured behavioral and non-behavioral CVD risk factors) to the observed association between job strain/insecurity and incident CVD using the formula: (HR_base model_ – HR_adjusted model_)/ (HR_base model_ –1)x100% [26]. All probability values were based on two-tailed tests. Statistical analyses were performed using SAS v. 9.2.

**Table 3:** Hazard rate (HR) and 95% confidence interval of cardiovascular disease by job insecurity (N = 22086).

	Job Secure	Job Insecure
	N = 17799	N = 4287
**Total CVD**		
Events (Total N = 661)	517	144
Model 1 †	1.00	**1.23 (1.02, 1.48)**
Model 2 ‡	1.00	1.19 (0.99, 1.43)
**Myocardial infarction**		
Events (Total N = 170)	129	41
Model 1	1.00	1.39 (0.98, 1.97)
Model 2	1.00	1.35 (0.95, 1.92)
**Ischemic stroke**		
Events (Total N = 163)	134	29
Model 1	1.00	0.97 (0.65, 1.45)
Model 2	1.00	0.94 (0.63, 1.40)
**Coronary revascularization**#		
Events (Total N = 440)	344	96
Model 1	1.00	1.22 (0.97, 1.53)
Model 2	1.00	1.19 (0.95, 1.49)
**Cardiovascular death**		
Events (Total N = 52)	39	13
Model 1	1.00	1.52 (0.81, 2.85)
Model 2	1.00	1.41 (0.75, 2.65)

95% confidence intervals shown in parentheses.

† Model 1 is adjusted for age; race; and study drug randomization.

‡ Model 2 is adjusted for covariates in Model 1, in addition to education (<2 y health profession education; 2-<4 y health professional education; bachelor’s degree; master’s degree; doctorate); income (<$19,000; $20,000–29,999; $30,000–39,999; $40,000–49,999; $50,000–99,999; ≥$100,000).

§ Coronary revascularization includes coronary artery bypass grafting and percutaneous transluminal coronary angioplasty.

## Results

### Baseline Characteristics

**Table 1** presents the baseline characteristics of the study participants, stratified by (1) job strain category, and (2) job insecurity status. Following Karasek’s model of job strain, 23.8% of women had low job strain, while 34.4% had passive jobs, 21.4% had active jobs, and 20.4% had high job strain. Approximately one-fifth of the sample (19.4%) reported job insecurity, and it was most common among women in high strain jobs (32.2%). The mean age of the sample was 57.2 years. **Figure 1** shows the distribution of professional education level according to job strain and insecurity categories. Across categories of job strain, significant variations were noted in the prevalence of hypertension, hypercholesterolemia, depressive/anxious symptoms, physical activity, alcohol intake, educational attainment, median household income, employment status, and marital status (all p-values <0.001; see **Table 1**). For the majority of these characteristics, women in the passive and high strain categories had higher levels of risk factors relative to women in the low strain and active jobs categories. Compared to women who reported secure jobs, women reporting insecure jobs were more likely to have a higher-risk demographic and health profile for nearly every characteristic that was assessed (all p-values <0.05).

### Job Strain and CVD

Over the 10 years of follow-up, 661 women experienced a cardiovascular event; **Table 2** shows adjusted rate ratios (RR) for the relationship between job strain categories and total incident CVD, as well as specific endpoints. After adjustment for age, race, and randomized study-drug assignment (Model 1), women in passive jobs, active jobs, and high strain categories had elevated risk for any CVD relative to women in the low strain category (1.37, 1.39, and 1.63, respectively; all p-values ≤0.01). In Model 2, which additionally adjusted for education and income, women in the active jobs and high strain categories continued to display significantly elevated risk for CVD (RR = 1.38 and 1.38, respectively; both p-values <0.05). Examination of individual CVD endpoints demonstrated associations between high job strain and elevated risk for MI (RR = 1.67, 95% CI = 1.04–2.70) and coronary revascularization (RR = 1.41, 95% CI = 1.05–1.90) relative to women who reported low strain. Because depressive/anxious symptoms and traditional CVD risk factors potentially lie on the causal pathway for CVD risk, we considered Model 2 to be the most clinically valid model.

In the subset of women with complete data on a more extensive range of covariates (N = 17,415; see **Table S4**), our findings for Model 1 and Model 2 remained relatively consistent with the results from the larger sample presented in Table 2, although the rate ratios for Model 1 and Model 2 were slightly more pronounced in this reduced sample. Additional adjustment for depressive/anxious symptoms (Model 3), and full adjustment for traditional CVD risk factors (Model 4) attenuated the majority of significant associations; however, women in the active jobs category maintained significantly elevated risk for total CVD (RR = 1.50, 95% CI = 1.13–1.99; p<0.001), and high job strain was associated with an elevated risk for MI (RR = 1.80, 95% CI = 1.02–3.21), relative to women who reported low job strain.

We further evaluated the extent to which traditional CVD risk factors and depressive/anxious symptoms might potentially mediate the observed relations between high strain and active jobs with elevated CVD risk. As shown in **Figure 2**, the relationships between high strain and active jobs and incident CVD were only minimally explained by the traditional CVD risk factors in our study. Depressive/anxious symptoms explained the highest proportion of the observed high strain-CVD association (21%). In contrast, depressive/anxious symptoms explained only 8% of the active jobs-incident CVD relationship.

**Table 3** presents adjusted RRs for models that examined job insecurity in relation to incident CVD outcomes. No statistically significant relationship was noted between job insecurity and 10 year total CVD risk (Model 2). Likewise, no significant associations were observed in models predicting outcome-specific CVD endpoints, although the direction of the rate ratios for all outcomes indicated excess risk for women reporting job insecurity.

In sensitivity analyses we examined dimensions of demand and control separately, with each dimension categorized into a dichotomous variable based on the median value for each scale. High demand was associated with total CVD (RR = 1.25, 95% CI = 1.07–1.47) after adjustment for age, race/ethnicity, study drug randomization, education and income, and that association remained significant in the fully adjusted model (Model 4; RR = 1.22, 95% CI = 1.02–1.47). We did not detect a significant association between high demand and incident CVD in models using a continuous demand score, or when demand was categorized in tertiles. Low control was associated with total CVD risk after adjustment for age, race/ethnicity, and study drug randomization (OR = 1.25, 95% CI = 1.07, 1.47), but this relationship was not maintained after control for education and income (OR = 1.07, 95% CI = 0.90, 1.26). We did not find evidence for interactions between continuous or categorical measures of demands and control, categorical measures of job strain and job insecurity, or between age or race/ethnicity with job strain or job insecurity in models of total CVD events (all p-values >0.05). Finally, tests of the proportional hazards assumption indicated that this assumption was not violated (e.g., Model 2 total CVD: job strain p-value = 0.45; job insecurity p-value = 0.62).

## Discussion

This 10 year prospective study of female health professionals revealed that women with active jobs (high demand, high control) and high strain (high demand, low control) were 38% more likely to experience a first CVD event relative to women reporting low job strain, adjusting age, race, study drug randomization, education, and income. In a sub-analysis that included women with complete data on a broader range of covariates, the association between high strain and CVD was not significant after adjustment for SES and traditional coronary risk factors, whereas women with active jobs were 50% more likely to experience a CVD event even after adjustment for these factors. These results suggest that women with high strain and active jobs potentially experience long-term vascular effects where high demand appears to be the critical factor. Job insecurity did not predict incident CVD, but was cross-sectionally associated with risk factors for CVD including smoking, physical inactivity, hypertension, diabetes, hypercholesterolemia, and BMI in bivariate analyses.

Our findings extend previous work on job strain by demonstrating a relationship between job strain at baseline and clinically-validated CVD end points in a large cohort of relatively healthy women. The results are compatible with prior studies comprised of predominantly male subjects [6], [8], [27], and with some studies involving women [15], [17]. For example, among 4191 Swedish women aged 20 to 64 reporting a combination of hectic and monotonous work, there was a 1.6-fold increase in hospitalization for MI (95% CI = 1.1–2.3) over 1 year of follow up [15]. Our findings are also consistent with a Swedish study of 49,259 women which found no relationship between job strain and incident stroke [28].

The association between active jobs and incident total CVD was not expected although recent work demonstrates a relationship with cerebrovascular disease [12], [20]. Among 48,361 women in Finland, active jobs were associated with increased risk of incident cerebrovascular disease (OR = 2.32, 95% CI = 1.3–4.1) [20]. In the Framingham Offspring Study which included 1,328 women aged 18–77, women with active jobs had a 2.8-fold increased risk of CHD (95% CI = 1.1–7.2) over 10 years, relative to women with low job strain [12]. This association between active jobs and CHD was not observed for men in the same study. These authors hypothesized that this association may be due to enduring resistance and gender-discrimination experienced by women in positions of authority and control. Of note, our findings on the relationship between job strain and CVD differ from those of some other studies [16], [29], including the Nurses Health Study (NHS) which found no increase in incidence of CHD associated with job strain over 4 years [16]. However, compared to the NHS, our study has a longer follow-up, and more variability in occupation type and associated work conditions.

Our observation of no relationship between job insecurity and 10 year incident CVD risk is in contrast with our a priori hypothesis that job insecurity would positively predict CVD risk, and conflicts with some prior research [19]. For example, in a 5-year study of 3413 middle-aged British female civil servants, Ferrie and colleagues [19] found a 1.6-fold increase in risk of coronary ischemia in women who anticipated organizational change relative to those who did not. Additionally, in the NHS, there was a relationship between job insecurity and 2 year coronary heart disease risk; however, this association no longer held at 4 years of follow-up [18]. In exploratory analyses (data not shown), we examined whether job insecurity was related to elevated short term CVD risk in our sample: we did not find evidence for a significant association after 2 or 5 years of follow-up.

From a mechanistic perspective, job strain may influence risk of CVD indirectly through behavioral responses such as smoking [30] and depression [31], or directly via physiological stress processes resulting in hypertension [32] or the metabolic syndrome [33]. At the biological level, chronic stress results in repeated activation of the physiological stress response causing dysregulation of the hypothalamic pituitary adrenal axis and autonomic nervous system. These disruptions may lead to a persistent elevation of blood pressure, destabilization of atherosclerotic plaques, up-regulation of cytokine expression, enhancement of cortisol secretion resulting in endothelial dysfunction, insulin resistance and other metabolic as well as hemodynamic perturbations, all critical determinants of heightened CVD risk [7], [27].

Our data show that job strain and insecurity have cross-sectional relationships with CVD risk factors and unhealthy behavior, but that only job strain is associated with long-term outcome among women in our cohort. These data also demonstrate that only between 11–26% of the job strain-CVD relationship could be explained by measured risk factors with depressive/anxious symptoms being the largest contributor to this percentage. This observation is intriguing because although psychological stress is a strong predictor of CVD risk, it seems that traditional behavioral and non-behavioral CVD risk factors only account for a small proportion of the CVD risk ascribed to psychological stress. Thus, there remains great need to identify other potential mediators of the risk relationship including examination of the role of the co-occurring psychosocial stressors, environmental factors, and the role of social buffering.

The results of this 10 year follow-up study must be considered within the context of several limitations. First, our cohort consisted of predominantly white female health professionals. These analyses should be repeated in samples with greater racial/ethnic variation, and occupational diversity, particularly since the health industry tends to maintain better relative job stability compared to other job categories/types during economic instability. Second, job strain and job insecurity were measured at a single time-point. Some work has shown that repeated measures of job-related stressors might be important for monitoring the chronicity of exposure because single-time exposure measures may underestimate the relationship between chronic strain and CVD [34]. Third, job insecurity was assessed using a single-item measure; however, our measure of job insecurity is consistent with measures utilized in prior research [18] which facilitates comparison. Fourth, unmeasured personal characteristics, work-related factors, and other unmeasured confounders may influence reports of work strain and certain CVD risk factors. Fifth, it is possible that healthier women participated in the work force longer (and thus were eligible for the work stress items at Year 5); however, exclusion of less healthy women would lead to an underestimation of the effects of work strain on CVD risk. Related, 4671 women (21.1%) did not have complete data on the traditional CVD risk factor variables utilized in Models 3 and 4; we minimize the implications of this missing data by presenting Models 1 and 2 using the largest available sample, and replicating these models with the reduced sample to facilitate comparison. Finally, it is possible that we did not find a statistically significant association between job strain and CVD death due to a lower number of CVD deaths over the follow-up period relative to other outcomes (n = 52).

To conclude, given the limited number of studies on this topic that have included women, it will be important for additional studies to continue to investigate associations between job stress and CVD particularly in multi-ethnic female populations. Moreover, studies evaluating the impact of interventions to improve psychosocial work environment characteristics are scarce [35] and might be occupation and population specific. Our findings suggest the need to develop interventions to improve psychosocial characteristics of the work environment since this may have long-term benefits for cardiovascular health in women. Similarly, research is needed to develop and validate employee work models that minimize work stress. From a clinical perspective, it may be useful for health professionals to screen patients for psychosocial stressors and to connect individuals to resources for healthy stress management.

## Supporting Information

Figure S1 **Flow Chart of Sample.**
(DOC)

Table S1 **Comparison of baseline characteristics for participants in main analysis (N = 22,086) and participants excluded from main analysis due to missing work stress questions (N = 10,833) or critical covariates for Models 1 and 2 (N = 1748).**
(DOC)

Table S2 **Comparison of baseline characteristics for participants included in sub-analysis (N = 17,415) and participants excluded from sub-analysis due to missing data (N = 4671).**
(DOC)

Table S3 **Items used to create job strain categories.**
(DOC)

Table S4 **Hazard rate (HR) and 95% confidence interval of cardiovascular disease by job strain, sample restricted to women with completed data on extensive set of potential mediators and confounders (N = 17415).**
(DOC)

Table S5 **Cardiovascular risk factors and other characteristics according to job strain and job insecurity for subset of women with complete data on traditional behavioral and non-behavioral CVD risk factors (N = 17415).**
(DOC)
