# Supplementary figures and images for: Job Strain, Job Insecurity, and Incident Cardiovascular Disease in the Women’s Health Study: Results from a 10-Year Prospective Study
Source: PLoS One. 2012 Jul 18;7(7):e40512. doi: 10.1371/journal.pone.0040512 (PMC3399852; doi:10.1371/journal.pone.0040512)

**Figure S1.** Flow Chart of Sample


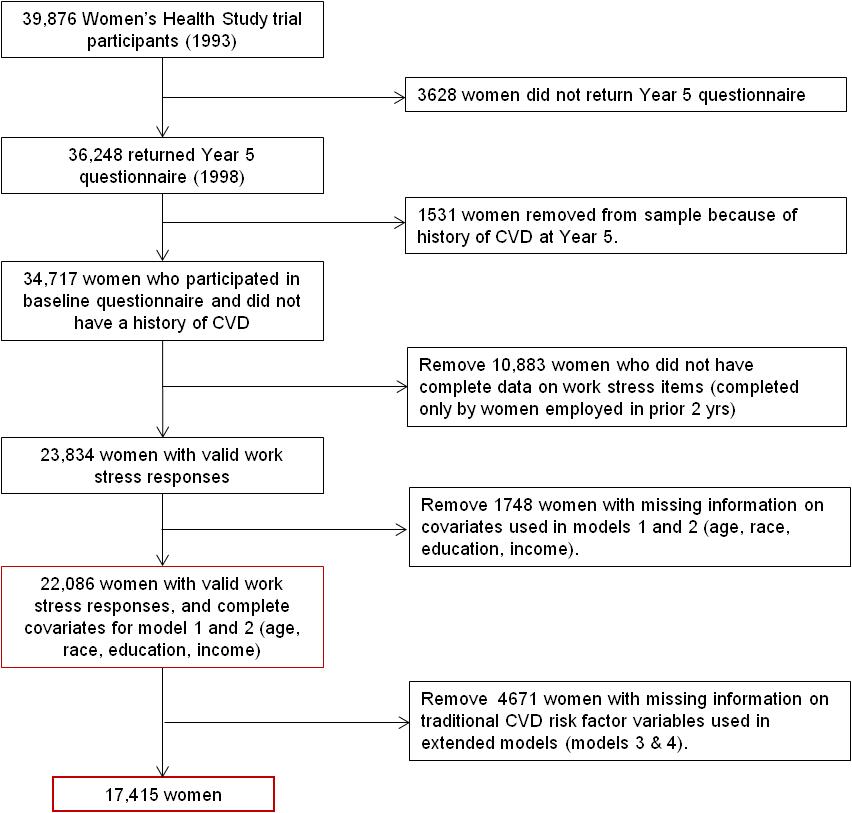

Supplement: Figure S1 — Flow Chart of Sample. (DOC) [file pone.0040512.s001.doc]
