# Supplementary material for: Job Strain, Job Insecurity, and Incident Cardiovascular Disease in the Women’s Health Study: Results from a 10-Year Prospective Study
Source: PLoS One. 2012 Jul 18;7(7):e40512. doi: 10.1371/journal.pone.0040512 (PMC3399852; doi:10.1371/journal.pone.0040512)
Supplement: Table S1 — Comparison of baseline characteristics for participants in main analysis (N = 22,086) and participants excluded from main analysis due to missing work stress questions (N = 10,833) or critical covariates for Models 1 and 2 (N = 1748). (DOC) [file pone.0040512.s002.doc]

| **Table S1:** Comparison of baseline characteristics for participants in main analysis (N=22,086) and participants excluded from main analysis due to missing work stress questions (N=10,833) or critical covariates for Models 1 and 2 (N=1748) | | | | | |
| --- | --- | --- | --- | --- | --- |
|  | Included in analysis | Excluded due to missing work stress questions/not working |  | Excluded due to missing on education, income or race |  |
|  | (N=22086) | (N=10,883) |  | (N=1748) |  |
|  | % or Mean (N) | % or Mean (N) | p-value^1^ | % or mean (N) | p-value^1^ |
| Mean Age, years | 57.25 (22086) | 64.57 (10883) | <.0001 | 57.54 (1748) | 0.02 |
| Hypertension (%) | 37.99 (8390) | 50.98 (5548) | <.0001 | 37.24 (651) | 0.54 |
| Diabetes mellitus (%) | 3.92 (866) | 5.49 (597) | <.0001 | 2.80 (49) | 0.02 |
| Hypercholesterolemia (%) | 42.13 (9304) | 52.60 (5725) | <.0001 | 43.19 (755) | 0.39 |
| Depressive/anxious symptoms (mean) | 10.52 (21503) | 9.54 (10176) | <.0001 | 10.60 (1689) | 0.42 |
| Body mass index (kg/m^2^) (mean) | 27.06 (22002) | 26.58 (10700) | <.0001 | 26.63 (1744) | .001 |
| Physical activity (%) |  |  |  |  |  |
| Rare/never | 36.50 (8057) | 41.03 (4463) | <.0001 | 36.84 (644) | 0.89 |
| < 1 time a week | 20.77 (4584) | 17.57 (1911) |  | 21.28 (372) |  |
| 1-3 times a week | 32.36 (7144) | 29.35 (3193) |  | 31.52 (551) |  |
| 4 times a week | 10.37 (2290) | 12.05 (1311) |  | 10.35 (181) |  |
| 1+ Alcoholic Drink/Day (%) | 10.56 (2294) | 12.12 (12.90) | <.0001 | 9.65 (166) | 0.24 |
| Current Smoker (%) | 10.68 (2325) | 9.59 (1025) | 0.002 | 10.37 (179) | 0.69 |
| Education (%) |  |  |  |  |  |
| <2 y health prof. education | 12.49 (2758) | 15.56 (1662) | <.0001 | 13.58 (186) | 0.04 |
| 2-<4 y of health prof. education | 39.98 (8829) | 49.55 (5291) |  | 42.92 (588) |  |
| BS degree | 24.78 (5472) | 20.58 (2197) |  | 23.80 (326) |  |
| MS degree | 17.33 (3828) | 10.44 (1115) |  | 15.26 (209) |  |
| Doctorate | 5.43 (1199) | 3.87 (413) |  | 4.45 (61) |  |
| Household Income (%) |  |  |  |  |  |
| <$19,000 | 2.88 (635) | 10.74 (1087) | <.0001 | 3.40 (18) | 0.11 |
| $20,000-29,999 | 7.51 (1659) | 15.25 (1543) |  | 9.62 (51) |  |
| $30,000-39,999 | 12.59 (2861) | 17.36 (1757) |  | 13.21 (70) |  |
| $40,000-49,999 | 16.97 (3748) | 15.13 (1531) |  | 18.49 (98) |  |
| $50,000-99,999 | 46.22 (10209) | 30.66 (3102) |  | 45.28 (240) |  |
| >$100,000 | 13.47 (2974) | 10.86 (1099) |  | 10.00 (53) |  |
| Employment status (%) |  |  |  |  |  |
| Employed full time or part time | 89.59 (19710) | 19.34 (2030) | <.0001 | 89.47 (1555) | 0.82 |
| Full time home maker/ Retired/ Not employed | 10.04 (2209) | 78.53 (8243) |  | 10.07 (175) |  |
| Disabled | 0.36 (80) | 2.12 (223) |  | 0.46 (8) |  |
| Marital status (%) |  |  |  |  |  |
| Single | 5.96 (1276) | 5.04 (532) | <.0001 | 7.16 (121) | <.0001 |
| Currently married | 74.25 (15898) | 73.96 (7807) |  | 77.46 (1309) |  |
| Divorced or separated | 15.42 (3301) | 10.00 (1056) |  | 11.24 (190) |  |
| Widowed | 4.37 (936) | 10.99 (1160) |  | 4.14 (70) |  |
| Parent history of MI before 60 yrs (%) | 13.47 (2671) | 11.40 (1118) | <.0001 | 12.32 (194) | 0.20 |
| ^1^Comparison to those included in analysis (N=22,086) | | | | | |
