# Supplementary material for: Job Strain, Job Insecurity, and Incident Cardiovascular Disease in the Women’s Health Study: Results from a 10-Year Prospective Study
Source: PLoS One. 2012 Jul 18;7(7):e40512. doi: 10.1371/journal.pone.0040512 (PMC3399852; doi:10.1371/journal.pone.0040512)
Supplement: Table S2 — Comparison of baseline characteristics for participants included in sub-analysis (N = 17,415) and participants excluded from sub-analysis due to missing data (N = 4671). (DOC) [file pone.0040512.s003.doc]

| **Table S2:** Comparison of baseline characteristics for participants included in sub-analysis (N=17,415) and participants excluded from sub-analysis due to missing data (N=4671) | | | |
| --- | --- | --- | --- |
|  | Included in analysis(N=17415) | Excluding due to missing data required for Models 3 and 4 (N=4671) |  |
|  | % or Mean (N) | % or Mean (N) | p-value |
| Job Insecure (%) | 19.18 (3340) | 20.27 (947) | 0.09 |
| Job Strain Category (%) |  |  |  |
| Low Strain | 23.89 (4161) | 23.55 (1100) | 0.72 |
| Passive | 34.39 (5989) | 34.58 (1615) |  |
| Active | 21.45 (3736) | 20.98 (980) |  |
| High Strain | 20.26 (3529) | 20.89 (976) |  |
| Mean Age, years | 57.33 (17415) | 56.94 (4671) | <.0001 |
| Hypertension (%) | 37.81 (6585) | 38.64 (1805) | 0.30 |
| Diabetes mellitus (%) | 3.97 (691) | 3.75 (175) | 0.49 |
| Hypercholesterolemia (%) | 41.99 (7313) | 42.62 (1991) | 0.44 |
| Depressive/anxious symptoms (mean) | 10.49 (17415) | 10.66 (4088) | 0.01 |
| Body mass index (kg/m^2^) (mean) | 27.03 (17415) | 27.19 (4587) | 0.07 |
| Physical activity (%) |  |  |  |
| Rare/never | 36.37 (6333) | 37.00 (1724) | 0.73 |
| < 1 time a week | 20.71 (3606) | 20.99 (978) |  |
| 1-3 times a week | 5665 (32.53) | 31.74 (1479) |  |
| 4 times a week | 10.40 (1811) | 10.28 (479) |  |
| 1+ Alcoholic Drink/Day (%) | 10.36 (1805) | 11.35 (489) | 0.06 |
| Current Smoker (%) | 10.79 (1879) | 10.26 (446) | 0.31 |
| Education (%) |  |  |  |
| <2 y health prof. education | 12.34 (2149) | 13.04 (609) | 0.01 |
| 2-<4 y of health prof. education | 39.84 (6939) | 40.46 (1890) |  |
| BS degree | 24.67 (4297) | 25.16 (1175) |  |
| MS degree | 17.44 (3038) | 16.91 (790) |  |
| Doctorate | 5.70 (992) | 4.43 (207) |  |
| Household Income (%) |  |  |  |
| <$19,000 | 2.87 (499) | 2.91 (136) | 0.20 |
| $20,000-29,999 | 7.66 (1334) | 6.96 (325) |  |
| $30,000-39,999 | 12.83 (2235) | 13.40 (626) |  |
| $40,000-49,999 | 17.21 (2997) | 16.08 (751) |  |
| $50,000-99,999 | 46.08 (8025) | 46.76 (2184) |  |
| >$100,000 | 13.35 (2325) | 13.89 (649) |  |
| Employment status (%) |  |  |  |
| Employed full time or part time | 89.46 (15579) | 90.12 (4131) | 0.10 |
| Full time home maker/ Retired/ Not employed | 10.21 (1778) | 9.40 (431) |  |
| Disabled | 0.33 (58) | 0.48 (22) |  |
| Marital status (%) |  |  |  |
| Single | 5.87 (1023) | 6.33 (253) | 0.22 |
| Currently married | 74.17 (12916) | 74.62 (2982) |  |
| Divorced or separated | 15.47 (2694) | 15.19 (607) |  |
| Widowed | 4.49 (782) | 3.85 (154) |  |
| Parent history of MI before 60 yrs (%) | 13.49 (2350) | 13.29 (321) | 0.78 |
|  |  |  |  |
