# Supplementary material for: Job Strain, Job Insecurity, and Incident Cardiovascular Disease in the Women’s Health Study: Results from a 10-Year Prospective Study
Source: PLoS One. 2012 Jul 18;7(7):e40512. doi: 10.1371/journal.pone.0040512 (PMC3399852; doi:10.1371/journal.pone.0040512)
Supplement: Table S3 — Items used to create job strain categories. (DOC) [file pone.0040512.s004.doc]

| **Table S3.** Items used to create job strain categories |
| --- |
| ***Job demands*** |
| 1. My job requires working very fast |
| 1. My job requires working very hard |
| 1. I am not asked to do an excessive amount of work* |
| 1. I have enough time to get the job done* |
| 1. I am free from conflicting demands that others make* |
|  |
| ***Job Control*** |
| *Decision authority* |
| 1. My job allows me to make a lot of decisions on my own |
| 1. On my job, I have very little freedom to decide how I do my work* |
| 1. I have a lot of say about what happens on my job |
| *Skill utilization* |
| 1. My job requires that I learn new things |
| 1. My job involves a lot of repetitive work* |
| 1. My job requires me to be creative |
| 1. My job requires a high level of skill |
| 1. I get to do a variety of different things on my job |
| 1. I have an opportunity to develop my own special abilities |
| *Reverse scored |
