# Supplementary material for: Job Strain, Job Insecurity, and Incident Cardiovascular Disease in the Women’s Health Study: Results from a 10-Year Prospective Study
Source: PLoS One. 2012 Jul 18;7(7):e40512. doi: 10.1371/journal.pone.0040512 (PMC3399852; doi:10.1371/journal.pone.0040512)
Supplement: Table S4 — Hazard rate (HR) and 95% confidence interval of cardiovascular disease by job strain, sample restricted to women with completed data on extensive set of potential mediators and confounders (N = 17415). (DOC) [file pone.0040512.s005.doc]

| **Table S4.** Hazard rate (HR) and 95% confidence interval of cardiovascular disease by job strain, sample restricted to women with completed data on extensive set of potential mediators and confounders (N=17415) | | | | |
| --- | --- | --- | --- | --- |
|  | Low strain | Passive | Active | High strain |
|  | (low demand, high control) | (low demand, low control) | (high demand,high control) | (high demand, low control) |
|  | N=4161 | N=5989 | N=3736 | N=3529 |
| **Total CVD** |  |  |  |  |
| Events (Total N=519) | 89 | 208 | 107 | 115 |
| Model 1† | 1.00 | **1.44 (1.12, 1.84)** | **1.57 (1.18, 2.09)** | **1.67 (1.27, 2.21)** |
| Model 2‡ | 1.00 | 1.21 (0.94, 1.57) | **1.56 (1.17, 2.06)** | **1.40 (1.06, 1.86)** |
| Model 3§ | 1.00 | 1.19 (0.92, 1.53) | **1.51 (1.14, 2.01)** | 1.32 (0.99, 1.76) |
| Model 4| | | 1.00 | 1.22 (0.95, 1.58) | **1.50 (1.13, 1.99)** | 1.31 (0.98, 1.75) |
| **Myocardial infarction** |  |  |  |  |
| Events (Total N=134) | 20 | 54 | 26 | 34 |
| Model 1 | 1.00 | **1.69 (1.01, 2.83)** | 1.62 (0.90, 2.91) | **2.12 (1.22, 3.69)** |
| Model 2 | 1.00 | 1.50 (0.89, 2.54) | 1.61 (0.90, 2.90) | **1.88 (1.07, 3.30)** |
| Model 3 | 1.00 | 1.45 (0.86, 2.46) | 1.54 (0.86, 2.77) | 1.71 (0.96, 3.03) |
| Model 4 | 1.00 | 1.54 (0.91, 2.62) | 1.56 (0.86, 2.80) | **1.80 (1.02, 3.21)** |
| **Ischemic stroke** |  |  |  |  |
| Events (Total N=125) | 22 | 53 | 22 | 28 |
| Model 1 | 1.00 | 1.40 (0.85, 2.31) | 1.37 (0.76, 2.45) | 1.67 (0.95, 2.92) |
| Model 2 | 1.00 | 1.14 (0.68, 1.90) | 1.33 (0.73, 2.42) | 1.31 (0.74, 2.32) |
| Model 3 | 1.00 | 1.12 (0.67, 1.86) | 1.30 (0.72, 2.37) | 1.25 (0.70, 2.22) |
| Model 4 | 1.00 | 1.19 (0.71, 1.99) | 1.32 (0.73, 2.40) | 1.26 (0.70, 2.25) |
| **Coronary revascularization**# |  |  |  |  |
| Events (Total N=342) | 61 | 137 | 66 | 78 |
| Model 1 | 1.00 | **1.39 (1.03, 1.89)** | 1.39 (0.98, 1.97) | **1.64 (1.17, 2.29)** |
| Model 2 | 1.00 | 1.22 (0.89, 1.66) | 1.38 (0.97, 1.96) | **1.43 (1.01, 2.01)** |
| Model 3 | 1.00 | 1.18 (0.87, 1.62) | 1.34 (0.94, 1.90) | 1.33 (0.94, 1.89) |
| Model 4 | 1.00 | 1.22 (0.89, 1.67) | 1.32 (0.93, 1.87) | 1.32 (0.93, 1.88) |
| **Cardiovascular death** |  |  |  |  |
| Events (Total N=40) | 9 | 13 | 10 | 8 |
| Model 1 | 1.00 | 0.83 (0.35, 1.95) | 1.81 (0.72, 4.56) | 1.32 (0.50, 3.45) |
| Model 2 | 1.00 | 0.61 (0.25, 1.47) | 1.89 (0.75, 4.79) | 0.99 (0.37, 2.67) |
| Model 3 | 1.00 | 0.61 (0.25, 1.49) | 1.91 (0.75, 4.84) | 1.01 (0.37, 2.76) |
| Model 4 | 1.00 | 0.63 (0.26, 1.55) | 1.97 (0.77, 5.00) | 1.02 (0.37, 2.81) |
| 95% confidence intervals shown in parentheses. | | | | |
| †Model 1 is adjusted for age; race; and study drug randomization. | | | | |
| ‡Model 2 is adjusted for covariates in Model 1, in addition to education (<2 y health profession education; 2-<4 y health professional education; bachelor’s degree; masters degree; doctorate); income (<$19,000; $20,000-29,999; $30,000-39,999; $40,000-49,999; $50,000-99,999; >$100,000). | | | | |
| §Model 3 is adjusted for covariates in Model 2, in addition to depressive/anxious symptoms. | | | | |
| | |Model 4 is adjusted for covariates in Model 3, in addition to smoking (never, past, current); alcohol intake (rare, 1-3 drinks/mth, 1-6 drinks/mth, 1+ drinks/day); body mass index; history of hypertension, diabetes mellitus, and hypercholesterolaemia; physical activity (rare/never; <1 time a week; 1-3 times a week; 4 times a week); parental history of myocardial infarction before age of 60 years; marital status (single, currently married, divorced, widowed); current work status (full or part time; retired or full time home maker; disabled); menopausal status (premenopausal, postmenopausal, biologically uncertain, unclear/subject unsure). | | | | |
| #Coronary revascularization includes coronary artery bypass grafting and percutaneous transluminal coronary angioplasty. | | | | |
