# Supplementary material for: Job Strain, Job Insecurity, and Incident Cardiovascular Disease in the Women’s Health Study: Results from a 10-Year Prospective Study
Source: PLoS One. 2012 Jul 18;7(7):e40512. doi: 10.1371/journal.pone.0040512 (PMC3399852; doi:10.1371/journal.pone.0040512)
Supplement: Table S5 — Cardiovascular risk factors and other characteristics according to job strain and job insecurity for subset of women with complete data on traditional behavioral and non-behavioral CVD risk factors (N = 17415). (DOC) [file pone.0040512.s006.doc]

| **Table S5.** Cardiovascular risk factors and other characteristics according to job strain and job insecurity for subset of women with complete data on traditional behavioral and non-behavioral CVD risk factors (N=17415) | | | | | | | | | |
| --- | --- | --- | --- | --- | --- | --- | --- | --- | --- |
| **Job Characteristics** | **Full Sample** | **Job Strain Categories** | | | | | **Job Insecurity** | | |
|  |  | **Low strain** | **Passive** | **Active** | **High strain** | *p*-value | **Insecure** | **Secure** | *p*-value |
|  |  | (low demand, high control) | (low demand, low control) | (high demand, high control) | (high demand, low control) |  |  |  |  |
| Full sample, N (%) | -- | 4161 (23.9) | 5989 (34.4) | 3736 (21.4) | 3529 (20.3) |  | 3340 (19.2) | 14075 (80.8) |  |
| Job insecure (%) | -- | 11.5 | 18.2 | 17.7 | 31.5 | <0.001 | -- | -- | -- |
| Age, years (Mean, SD) | 57.3 (5.2) | 57.4 (5.1) | 58.4 (5.8) | 56.1 (4.2) | 56.8 (4.7) | <0.001 | 57.0 (5.0) | 57.4 (5.3) | <0.001 |
| Education (%) |  |  |  |  |  |  |  |  |  |
| <2 y health prof. education | 12.3 | 5.9 | 18.1 | 6.1 | 16.8 | <0.001 | 14.3 | 11.9 | <0.001 |
| 2-<4 y of health prof. education | 39.8 | 31.3 | 44.6 | 34.6 | 47.4 |  | 39.9 | 39.8 |  |
| BS degree | 24.7 | 27.3 | 23.0 | 26.4 | 22.6 |  | 24.7 | 24.7 |  |
| MS degree | 17.4 | 26.6 | 11.7 | 23.1 | 10.5 |  | 17.3 | 17.5 |  |
| Doctorate | 5.7 | 8.9 | 2.6 | 9.9 | 2.8 |  | 3.8 | 6.2 |  |
| Household Income (%) |  |  |  |  |  |  |  |  |  |
| <$19,000 | 2.9 | 1.5 | 4.9 | 1.0 | 3.1 | <0.001 | 3.8 | 2.6 | <0.001 |
| $20-29,999 | 7.7 | 5.1 | 11.0 | 4.0 | 9.0 |  | 9.4 | 7.2 |  |
| $30-39,999 | 12.8 | 10.7 | 14.8 | 10.1 | 14.8 |  | 15.4 | 12.2 |  |
| $40 -49,999 | 17.2 | 15.5 | 18.4 | 15.3 | 19.3 |  | 18.8 | 16.8 |  |
| $50-99,999 | 46.1 | 49.5 | 41.4 | 52.2 | 43.6 |  | 41.8 | 47.1 |  |
| >$100,000 | 13.4 | 17.7 | 9.6 | 17.5 | 10.3 |  | 10.8 | 14.0 |  |
